# Supplementary material for: Neuropelveology for Endometriosis Management: A Systematic Review and Multilevel Meta-Analysis
Source: J Clin Med. 2024 Aug 9;13(16):4676. doi: 10.3390/jcm13164676 (PMC11355340; doi:10.3390/jcm13164676)
Supplement: Supplementary file 1 [file jcm-13-04676-s001.zip › jcm-3080056-supplementary.pdf]

Search strategy of study

5 "(((""Endometriosis""[Mesh]) OR ((((Endometriosis[Title/Abstract]) OR (Endometrioses[Title/Abstract])) OR (Endometrioma[Title/Abstract])) OR (Endometriomas[Title/Abstract]))) AND ((""Endometriosis""[Mesh]) OR ((((Endometriosis[Title/Abstract]) OR (Endometrioses[Title/Abstract])) OR (Endometrioma[Title/Abstract])) OR (Endometriomas[Title/Abstract])))) AND (((((((((((((((((((((Neuropelveology[Title/Abstract]) OR (superior & inferior hypogastric plexus[Title/Abstract])) OR (hypogastric plexus[Title/Abstract])) OR (The iliohypogastric nerve[Title/Abstract])) OR (Autonomic nerves of pelvis[Title/Abstract])) OR (Vesicle plexus[Title/Abstract])) OR (Rectal plexus[Title/Abstract])) OR (sacral plexus[Title/Abstract])) OR (coccygeal plexus[Title/Abstract])) OR (lumbar plexus[Title/Abstract])) OR (sciatic nerve[Title/Abstract])) OR (Pudendal nerve[Title/Abstract])) OR (Coccygeal plexus[Title/Abstract])) OR (ilioinguinal nerve[Title/Abstract])) OR (pelvic nerve[Title/Abstract])) OR (neurosurgical techniques[Title/Abstract])) OR (decompression[Title/Abstract])) OR (neurolysis[Title/Abstract])) OR (reconstruction[Title/Abstract])) OR (nerve resection[Title/Abstract])) OR (Nerve Ablation[Title/Abstract])) OR (pelvic neurofunctional surgery[Title/Abstract]))" Most Recent "(""Endometriosis""[MeSH Terms] OR (""Endometriosis""[Title/Abstract] OR ""Endometrioses""[Title/Abstract] OR ""Endometrioma""[Title/Abstract] OR ""Endometriomas""[Title/Abstract])) AND (""Endometriosis""[MeSH Terms] OR (""Endometriosis""[Title/Abstract] OR ""Endometrioses""[Title/Abstract] OR ""Endometrioma""[Title/Abstract] OR ""Endometriomas""[Title/Abstract])) AND (""Neuropelveology""[Title/Abstract] OR ((""superior""[All Fields] OR ""superior s""[All Fields] OR ""superiorities""[All Fields] OR ""superiority""[All Fields] OR ""superiors""[All Fields]) AND ""inferior hypogastric plexus""[Title/Abstract]) OR ""hypogastric plexus""[Title/Abstract] OR (""iliohypogastric nerve""[Title/Abstract]) OR ((""autonomic pathways""[MeSH Terms] OR (""autonomic""[All Fields] AND ""pathways""[All Fields]) OR ""autonomic pathways""[All Fields] OR (""autonomic""[All Fields] AND ""nerves""[All Fields]) OR ""autonomic nerves""[All Fields]) AND ""of pelvis""[Title/Abstract]) OR ((""vesicle""[All Fields] OR ""vesicle s""[All Fields] OR ""vesicles""[All Fields]) AND ""plexus""[Title/Abstract]) OR ""rectal plexus""[Title/Abstract] OR ""sacral plexus""[Title/Abstract] OR ""coccygeal plexus""[Title/Abstract] OR ""lumbar plexus""[Title/Abstract] OR ""sciatic nerve""[Title/Abstract] OR ""pudendal nerve""[Title/Abstract] OR ""coccygeal plexus""[Title/Abstract] OR ""ilioinguinal nerve""[Title/Abstract] OR ""pelvic nerve""[Title/Abstract] OR ""neurosurgical techniques""[Title/Abstract] OR ""decompression""[Title/Abstract] OR ""neurolysis""[Title/Abstract] OR ""reconstruction""[Title/Abstract] OR ""nerve resection""[Title/Abstract] OR ""nerve ablation""[Title/Abstract] OR (((""pelvics""[All Fields] OR ""pelvis""[MeSH Terms] OR ""pelvis""[All Fields] OR ""pelvic""[All Fields]) AND (""neurofunction""[All Fields] OR ""neurofunctional""[All Fields])) AND ""surgery""[Title/Abstract]))" 365 9:12:33
4 "(""Endometriosis""[Mesh]) OR ((((Endometriosis[Title/Abstract]) OR (Endometrioses[Title/Abstract])) OR (Endometrioma[Title/Abstract])) OR (Endometriomas[Title/Abstract]))" Most Recent """Endometriosis""[MeSH Terms] OR ""Endometriosis""[Title/Abstract] OR ""Endometrioses""[Title/Abstract] OR ""Endometrioma""[Title/Abstract] OR ""Endometriomas""[Title/Abstract]"
3 "(""Pelvic Pain""[Mesh]) OR (((Pain
2 (((((((((((((((((((((Neuropelveology[Title/Abstract]) OR (superior & inferior hypogastric plexus[Title/Abstract])) OR (hypogastric plexus[Title/Abstract])) OR (The iliohypogastric nerve[Title/Abstract])) OR (Autonomic nerves of pelvis[Title/Abstract])) OR (Vesicle plexus[Title/Abstract])) OR (Rectal plexus[Title/Abstract])) OR (sacral plexus[Title/Abstract])) OR (coccygeal plexus[Title/Abstract])) OR (lumbar plexus[Title/Abstract])) OR (sciatic nerve[Title/Abstract])) OR (Pudendal nerve[Title/Abstract])) OR (Coccygeal plexus[Title/Abstract])) OR (ilioinguinal nerve[Title/Abstract])) OR (pelvic nerve[Title/Abstract])) OR (neurosurgical techniques[Title/Abstract])) OR (decompression[Title/Abstract])) OR (neurolysis[Title/Abstract])) OR (reconstruction[Title/Abstract])) OR (nerve

resection[Title/Abstract])) OR (Nerve Ablation[Title/Abstract])) OR (pelvic neurofunctional surgery[Title/Abstract]) Most Recent """Neuropelveology""[Title/Abstract] OR ((""superior""[All Fields] OR ""superior s""[All Fields] OR ""superiorities""[All Fields] OR ""superiority""[All Fields] OR ""superiors""[All Fields]) AND ""inferior hypogastric plexus""[Title/Abstract]) OR ""hypogastric plexus""[Title/Abstract] OR (""iliohypogastric nerve""[Title/Abstract]) OR ((""autonomic pathways""[MeSH Terms] OR (""autonomic""[All Fields] AND ""pathways""[All Fields]) OR ""autonomic pathways""[All Fields] OR (""autonomic""[All Fields] AND ""nerves""[All Fields]) OR ""autonomic nerves""[All Fields]) AND ""of pelvis""[Title/Abstract]) OR ((""vesicle""[All Fields] OR ""vesicle s""[All Fields] OR ""vesicles""[All Fields]) AND ""plexus""[Title/Abstract]) OR ""rectal plexus""[Title/Abstract] OR ""sacral plexus""[Title/Abstract] OR ""coccygeal plexus""[Title/Abstract] OR ""lumbar plexus""[Title/Abstract] OR ""sciatic nerve""[Title/Abstract] OR ""pudendal nerve""[Title/Abstract] OR ""coccygeal plexus""[Title/Abstract] OR ""ilioinguinal nerve""[Title/Abstract] OR ""pelvic nerve""[Title/Abstract] OR ""neurosurgical techniques""[Title/Abstract] OR ""decompression""[Title/Abstract] OR ""neurolysis""[Title/Abstract] OR ""reconstruction""[Title/Abstract] OR ""nerve resection""[Title/Abstract] OR ""nerve ablation""[Title/Abstract] OR (((""pelvics""[All Fields] OR ""pelvis""[MeSH Terms] OR ""pelvis""[All Fields] OR ""pelvic""[All Fields]) AND (""neurofunction""[All Fields] OR ""neurofunctional""[All Fields])) AND ""surgery""[Title/Abstract])"
